# Mechanisms of halosulfuron methyl pesticide biosorption onto neem seeds powder

**DOI:** 10.1038/s41598-021-88929-7

**Published:** 2021-05-11

**Authors:** Atta ul Haq, Muhammad Saeed, Muhammad Usman, Ameer Fawad Zahoor, Muhammad Naveed Anjum, Tahir Maqbool, Shazia Naheed, Muhammad Kashif

**Affiliations:** 1 Department of Chemistry, Government College University Faisalabad, Faisalabad, Pakistan; 2 Department of Applied Chemistry, Government College University Faisalabad, Faisalabad, Pakistan

**Keywords:** Environmental sciences, Chemistry, Materials science

## Abstract

The current investigation was designed to remove halosulfuron methyl from aqueous media by means of neem seed powder (NSP) in batch modes. Characterizations of NSP were carried out by using EDX, SEM, FTIR, point of zero charge and surface analysis. Optimum operation conditions were scrutinized by studying the influence of different factors like solution pH, dose of NSP, contact time, initial halosulfuron methyl concentration and temperature. Result indicates the dependency of the removal of halosulfuron methyl on solution pH and maximal removal (54%) was achieved in acidic medium (i.e. pH 3.0). To identify the chemical surface of NSP, point of zero charge of NSP was determined and was found to be 6.5 which imply that the surface of NSP is positively charged below pH 6.6 and favored the anionic sorption. Kinetics of halosulfuron methyl were demonstrated well by pseudo second order due to highest R^2^ (0.99) owing to the nearness between experimental and calculated sorption capacities. Isotherm results imply that Langmuir was found to the principal model to explain the removal of halosulfuron methyl and maximum monolayer sorption capacity was determined to be 200 mg g^−1^. Thermodynamic parameters like ΔH°, ΔG° and ΔS° were calculated from van’t Hoff plot and were found negative which suggest that removal of halosulfuron methyl is exothermic and spontaneous at low temperature. These outcomes insinuate that neem seed power may be a valuable, inexpensive and ecofriendly biosorbent for the removal of pesticides.

## Introduction

Globalization as well as huge population pressure makes the pesticides as essential chemical agents for high quality food production. Pesticides have been identified in ground water which considered as primary fountain of drinking water worldwide. Therefore, the existence of pesticides water is of serious concern to authorities, society and potable water production agencies^1^. European Community Drinking Water Directive recommended an amount of 0.1 µg L^−1^ pesticide in drinking water. The presence of excess amounts of pesticides are highly toxic and cause various health problems like endocrine malfunction, interaction with androgen and estrogen receptors, and affect the role of thyroid whilst long-term effects of pesticides are not identified^2^.

Halosulfuron-methyl [methyl 3-chloro-5-(4,6-diethoxypyrimidi,-2-ylcarbamoylsulfamoyl)-1-methylpyrazole-4-carboxylate] is belong to sulfonylurea group of herbicide widely used throughout the world to control post-emergence broadleaf weed, sedge and grasses present in crops and vegetables^3^. The extensive usage of halosulfuron-methyl has caused the contamination of water of lakes and rivers through surface runoff and rain^4^. There are many traditional methods to treat wastewater containing pesticides such as ultraviolet oxidation, membrane techniques, chemical precipitation, ion exchange, electro dialysis and chemical coagulation photocatalysis^5–7^. However, due to serious demerits and limitations of these methods like high operative cost, low efficiency for diluted wastewaters and generation of hazardous wastes make them unsuitable for the removal pesticides from water^8–10^. Therefore, it is needed to investigate new methods and techniques for sequestration of pesticides present in water which are efficiently and effectively safeguard the ecosystem and our life^11^. Adsorption is considered as a promising alternative method to remove pesticides and other contaminants from wastewater owing to its eco-friendly, simplicity and inexpensive^12–14^. Sequestration of pollutants using biological materials by physiochemical process from dilute aqueous solution is known as biosorption^6^. A variety of biological materials have been used to remove various pollutants like raw plants, industrial waste, macroalgae, agricultural residues, sludge, animal materials etc.^15–19^. However, agro-based and forestry originating materials such as cork waste^20^, Marula seed husk^21^, olive tree pruning^22^, Phytolacca americana biomass^19^, maize stover^23^, cedar leaf^24^, peanut shells^25^ etc. have been extensively used recently for wastewater treatment. Neem (*Azadirachta indica*) belongs to Meliaceae family and mostly present in semitropical and tropical regions of the world such as Pakistan, Nepal, Bangladesh and India. Each part of the neem tree such as root, leaves, bark and seed are used in various medicines. Seeds of the neem can also be used as antiviral, antifungal and antiseptic agent. It has been predicted that fourteen million trees are available only in India which produces approximately 400,000 tones of neem seeds per year. A single neem tree of 15 years old may produces up to 20 kg fruit or 2 kg neem seeds. The neem seed consists of approximately 40–45% oil while the residual part is the cellular matrix^26^. According to literature different parts of neem tree like leaves, bark and husk have been investigated as biosorbent for heavy metals and dyes^27–37^. However, according to our knowledge seeds of the neem have not been used for the removal of pesticides. The aforementioned reasons and abundant availability of the neem seeds prompted us to utilize neem seeds for treatment of wastewater containing halosulfuron-methyl.

## Materials and methods

### Chemicals

All chemicals utilized in the current study were of analytical grade purity and were purchased from Merck. In the present study hydrochloric acid, sodium hydroxide, boric acid, phosphoric acid, nitric acid, and acetic acid were used without any further purification. Halosulfuron methyl (purity 75%) was purchased from local market in Faisalabad, Pakistan.

Halosulfuron methyl (C_13_H_15_ClN_6_O_7_S) is belong to sulfonylurea group of herbicide widely used to control post-emergence broadleaf weed, sedge and grasses in crops and vegetables. It is a white colored powder and soluble in water at 1650 mg L^−1^. It is stable under normal handling and storage conditions. The chemical structure of halosulfuron methyl is given below:

### Instruments

In the present study different instruments have been used for their respective purposes such as analytical balance (Sartorius-GC 2012 Germany) was used for weighing of the materials. Electrical grinder (Frtsch-Pulverisette 2 of Japan) was used to grind the materials while orbital shaker was used for shaking of biosorbent and pesticide solution. Electrical oven (Memmert Celsius 2005) and pH meter (WTW-Inolab 720 Series) were used for heating of biosorbent and determination of pH of solution respectively. The concentrations of the pesticide before and after biosorption studies were determined by the help of UV/Vis spectrophotometer. The scanning electron microscope (SEM-Model-JSM-5910, Japan JEOL) was used to study the morphology of biosorbent while elemental composition of the biosorbent was studied with Energy Dispersive X-ray (EDX-INCA 200 Oxford Instruments UK). To study the functional groups of biosorbent Fourier Transform Infrared spectrometer (Bruker ALPHA) was used while Surface Area Pore Size Analyzer (Model NOVA2200e, Quantachrome, USA) was used to study the surface analysis of biosorbent.

### Preparation of standard solution of halosulfuron methyl

Halosulfuron methyl solution (200 mg L^−1^) was initially prepared in acetone (0.1% v/v) by dissolving appropriate quantity of commercial (75%) halosulfuron methyl and dilute working solutions were then prepared in distilled water using dilution formula.

### Preparation of biosorbent

A sufficient amount of the neem seeds (*Azadirachta indica*) were collected from Faisalabad city. The seeds were then completely washed with tap water and lastly washed with distilled water. Then seeds were kept in daylight for 2 weeks and ground with the help of electrical grinder. The grinded materials were then passed through sieve of mesh size 355 µm. These materials were transferred in a beaker containing sufficient distilled water and finally kept overnight. Supernatant of beaker was decanted many times, then filtered and washed with distilled water numerous times. Ultimately, the materials were then transferred into a china dish and dried in oven at 120 °C. After drying, materials were collected in a bottle for next research work.

### Determination of point of zero charge (pHzpc)

Point of zero charge of NSP was determined by following salt addition method. A particular amount of NSP (100 mg) was added to 95 mL of NaCl (0.01 M) in beakers and the pH of the suspension was adjusted in the range of 2.0 to 10.0 with the help of 1.0 N HCl and 1.0 NaOH. The mixture was kept in water shaker bath for 6 h at ambient temperature. After 6 h of equilibration the final pH of the suspension was determined and plotted the difference (final pH − initial pH) against the initial pH. Point of zero charge was obtained at the intersection of initial pH with difference of pH (final pH − initial pH).

### Procedure for removal of halosulfuron methyl

The sorption experiments of Halosulfuron methyl using NSP were performed in batch system. The experiment was performed in 250 mL titration flask by incorporating a desirable quantity of NSP with 30 mL of halosulfuron methyl solution of particular concentration (33.33 mg L^−1^) at 30 °C. Mixture in flasks was then agitated for desirable interval of time at a fixed speed (150 revolutions per minute) in an orbital shaker. The mixture was then filtered and the concentration of halosulfuron methyl after sorption was determined at max = 240 nm by means of Ultraviolet/Visible spectrophotometer. The dependency removal of halosulfuron methyl on pH was studied by adjusting pH of flask contents in ranging of 3 to 10 with Britton Robinson buffer solution (NSP 0.01 g, halosulfuron methyl concentration 33.33 mg L^−1^ and stirring time 30 min) at 30 °C. The impact of dosage of sorbent was studied by changing amount of NSP in the ranging of 0.01 to 0.08 (gram) with particular concentration of halosulfuron methyl (33.33 mg L^−1^) at 30 °C and agitated for 30 min. The influence of contact time and kinetics of the sorption process were scrutinized by taking adequate concentration of halosulfuron methyl (33.33 mg L^−1^) and particular amount of NSP (0.01 g) at 30 °C and agitated for a time of contact ranging for 10 to 70 min. The influence of halosulfuron methyl initial concentration and isotherms were scrutinized by changing concentration of halosulfuron methyl from 33.33 to 133.33 mg L^−1^ with particular quantity of NSP (0.01 g) and stirring time (30 min) at 30 °C. The influence of temperature on removal of halosulfuron methyl was investigated in ranging of 30 to 80 °C while the keeping constant pH (3), time for agitation (30 min), amount of NSP (0.01 g) and initial concentration of halosulfuron methyl (33.33 mg L^−1^).

To calculate the quantity of halosulfuron methyl adsorbed per unit weight of NSP (q_t_) at a particular time (t) and percentage removal, following formulae were used:

1 $${q}_{t}= \frac{v}{m}\left[{C}_{o}- {C}_{t}\right]$$

2 $$Removal \; \left(\%\right)= \left[\frac{{C}_{O}- {C}_{t}}{{C}_{o}}\right]\times (100)$$

In these formulas C_o_ and C_t_ express the initial concentration and final concentration (mg L^−1^) of halosulfuron methyl, ‘m’ denotes the dosage of NSP (gram) and ‘V’ expresses volume (mL) of halosulfuron methyl solution.

### Regeneration study

The regeneration of contaminant-loaded biosorbent is sometime required for further biosorption study. Hence, recycling of the halosulfuron methyl-loaded NSP was conducted with different molar solutions of NaOH (1.0 M, 0.1 M and 0.01 M) because the removal of halosulfuron methyl is the minimum at higher pH. For this purpose, 1.0 g of halosulfuron methyl-loaded NSP was transferred in separate beakers and 50 mL of NaOH solution of different concentrations were in each beaker. The contents were stirred in an orbital shaker for about 1 h. The mixture was then filtered and the concentration of halosulfuron methyl after desorption was determined at max = 240 nm by means of Ultraviolet/Visible spectrophotometer.

## Results and discussion

### Characterizations of NSP

Neem seed powder (NSP) was characterized by EDX, SEM, surface area analysis and point of zero charge before and after removal of Halosulfuron methyl to confirm of removal of halosulfuron methyl from water media.

#### EDX analysis

**Figure 1:**
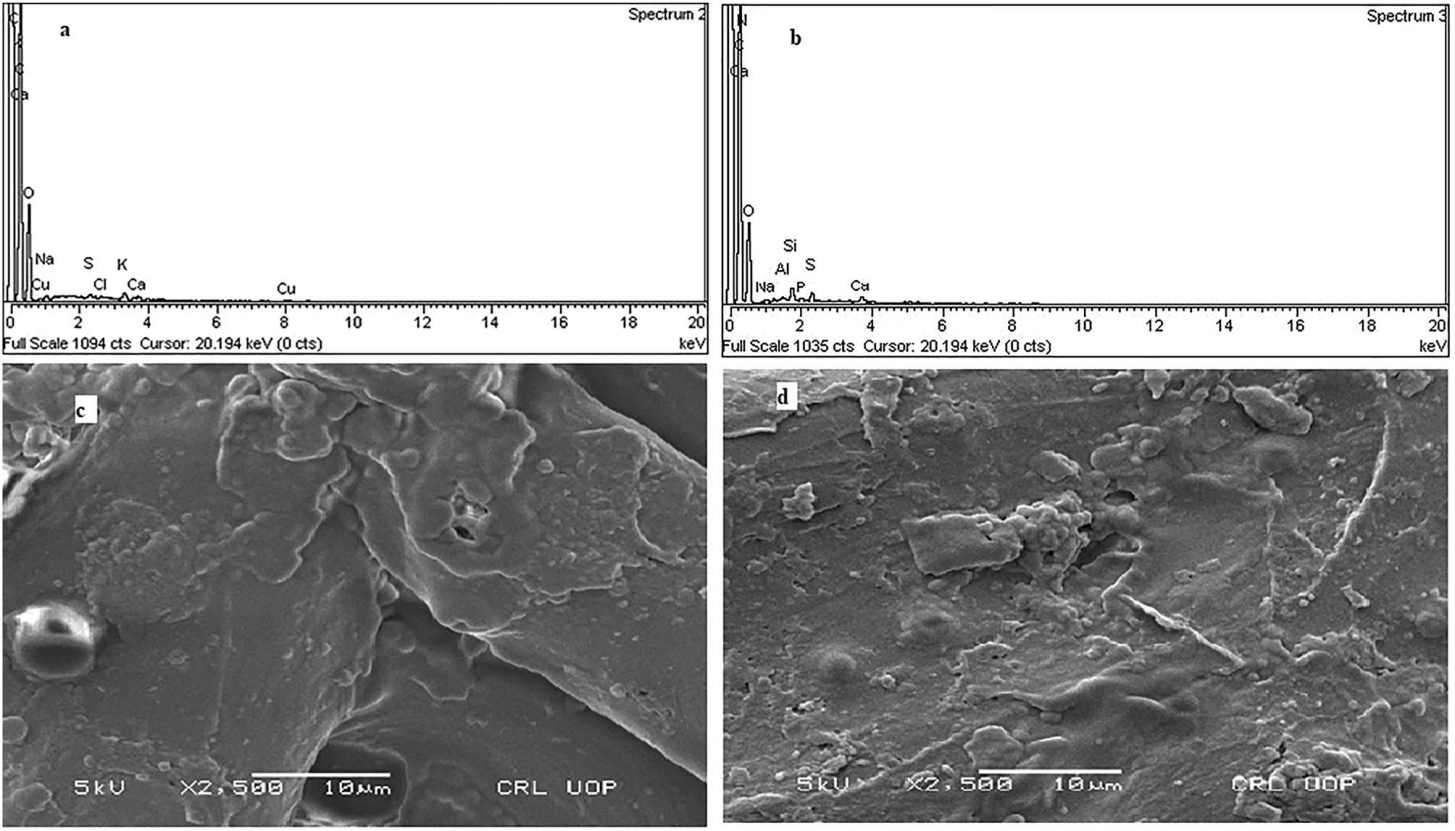
EDX of the NSP before removal of Halosulfuron methyl (**a**) EDX of the NSP after removal of Halosulfuron methyl (**b**) SEM of NSP before removal of Halosulfuron methyl (**c**) SEM of NSP after removal of Halosulfuron methyl (**d**).

To study variations on elementary level of NSP, EDX analysis was conducted before and after removal of halosulfuron methyl^38^. It can be demonstrated from the Fig. 1a that NSP consists of Cu, Ca, K, Cl, S Na, O and C before removal of Halosulfuron methyl. However, new peaks of N, Al, P and Si were appeared which indicates the variation in the elemental composition after removal of halosulfuron methyl as illustrated in Fig. 1b. Furthermore, the presence of the peak due to nitrogen after removal study proved that halosulfuron methyl was removed significantly because nitrogen atom is a part of halosulfuron methyl molecule.

#### SEM analysis

To assess any modification in surface morphology of NSP, SEM analysis was performed prior to and subsequent to halosulfuron methyl removal study. It is obvious from Fig. 1c that the surface of NSP is relatively smooth before removal of halosulfuron methyl but significant changes were observed after removal of halosulfuron methyl as illustrated in Fig. 1d and surface of NSP after removal of halosulfuron methyl became somewhat rough and irregular which indicates the removal of Halosulfuron methyl^39^.

#### Surface analysis

**Figure 2:**
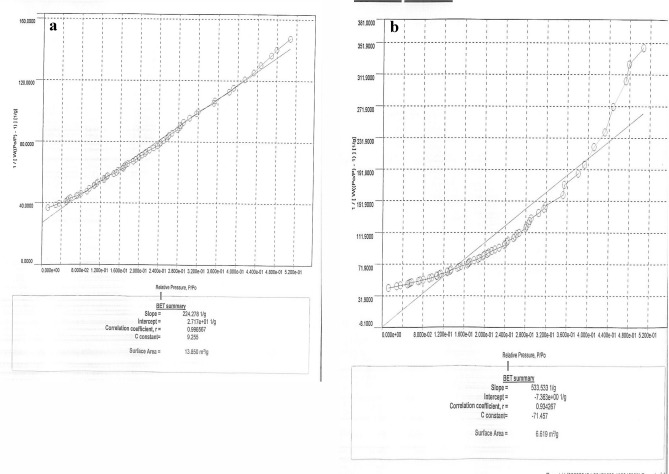
Nitrogen adsorption–desorption isotherm of NSP (**a**) before and (**b**) after biosorption of halosulfuron methyl.

Literature survey revealed that sorption ability of biomass is significantly altered by surface area and pore volume^40^. Hence, data of surface analysis were calculated using N_2_-adsorption isotherm prior to and subsequent to removal of halosulfuron methyl onto NSP. The results of surface analysis before and after biosorption of halosulfuron methyl are shown in Fig. 2a,b while the data is listed in Table 1. These outcomes demonstrate that surface area of NSP was greater before removal of halosulfuron methyl but was decreased after removal halosulfuron methyl which substantiates the process of removal of halosulfuron methyl from aqueous medium.

**Table 1:** Surface analysis of NSP using N_2_-adsorption isotherm.

Factor	Prior to removal of halosulfuron methyl	Subsequent to removal of halosulfuron methyl
Surface area (m^2^ g^−1^)	13.850	6.619
Pore volume (Å)	0.005	0.001
Pore radius (cc g^−1^)	15.035	15.008

#### Point of zero charge (pHzpc)

**Figure 3:**
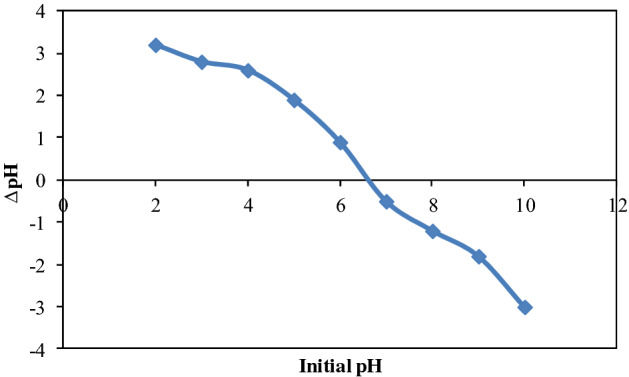
Point of zero charge of NSP.

Sorption mechanism can be easily recognized by knowing the magnitude of pHzpc of biomass^41^. The point of zero charge is the pH at which the surface of biomass has zero electrical charge density. The surface of biomass has positive charge below pHzpc while the surface becomes negatively charged above the pHzpc^42^. According to the literature cations removed more favorably at pH greater than pHzpc whereas anions removed more favorably at pH below pHzpc^43^. The pHzpc of NSP was assayed using salt addition method^44^. It can be depicted from the Fig. 3 that pHzpc of NSP is 6.5 which suggests that surface of NSP is negatively charged above 6.5 while positively charged below 6.5. Hence, low pH is more favorable for the removal of halosulfuron methyl and maximum removal of halosulfuron methyl was obtained from aqueous solution at low pH as depicted in Fig. 6.

#### FTIR analysis

**Figure 4:**
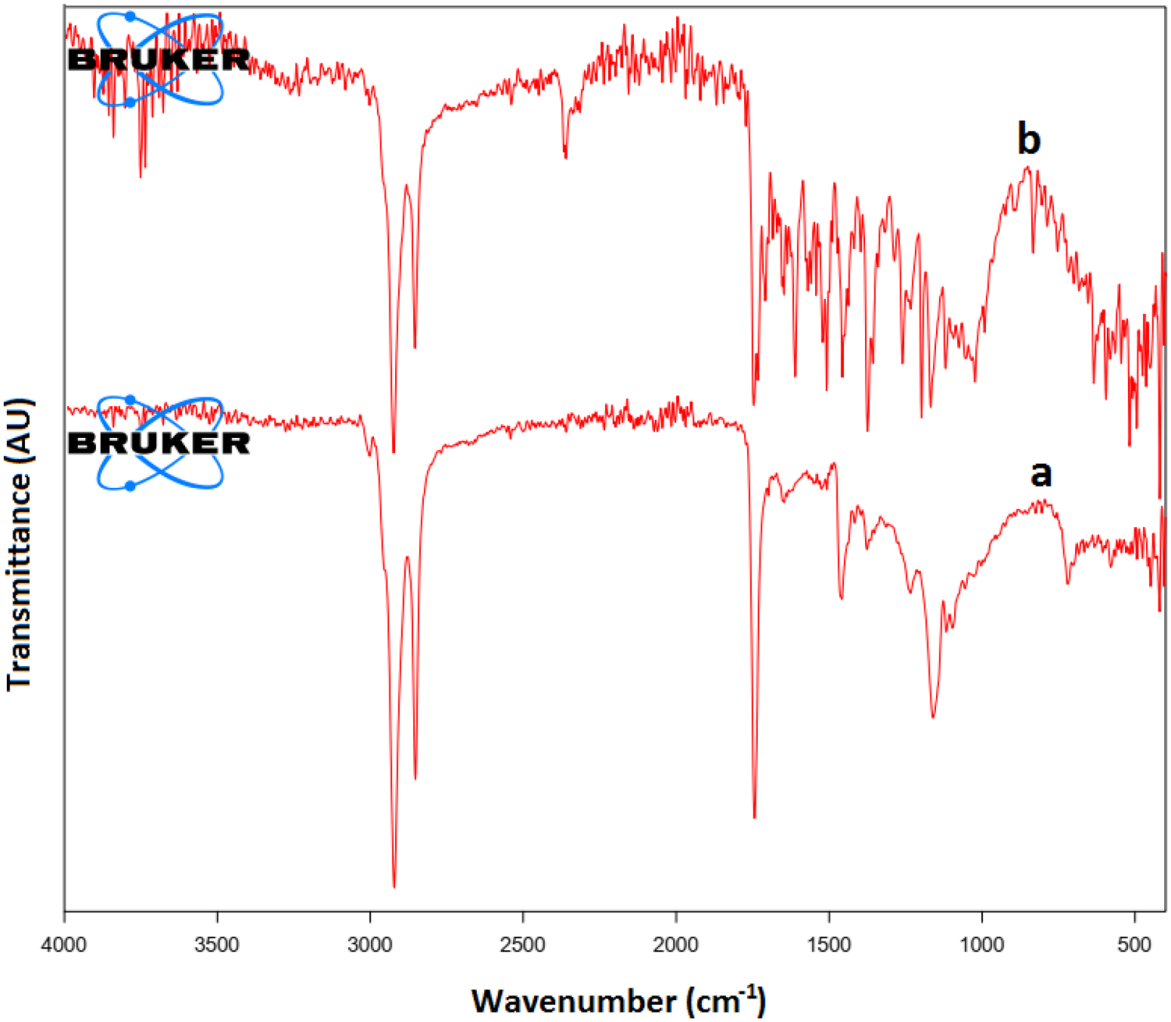
FTIR spectra of NSP before and after biosorption of halosulfuron methyl.

FTIR spectrum of the NSP before biosorption of halosulfuron methyl showed distinct peaks at 2921.54, 2852.31, 1743.72, 1458.37 and 1160.58 cm^−1^ (Fig. 4a) and each peak could be assigned to particular functional groups as mentioned in the literature. The intense two peaks at 2921.54, 2852.31 might be assigned to C‒H stretching vibrations of the alkyl group while the intense peak 1743.72 might be attributed to C=O stretching vibration of carbonyl group. Similarly the spectral peaks at 1458.37 and 1160.58 represent the C‒O stretching vibration of carboxylic acid and C‒O‒C stretching vibration of ether respectively. After biosorption some new peaks are appeared such as 3854.43, 2360.43, 1647.73, 1610.84, 1374.31 cm^−1^ (Fig. 4b) which indicates the complex nature of halosulfuron methyl pesticide. Similarly, the intensities of some peaks have changed and some peaks have sifted which indicates the biosorption of halosulfuron methyl pesticide.

### Effect of pH

**Figure 5:**
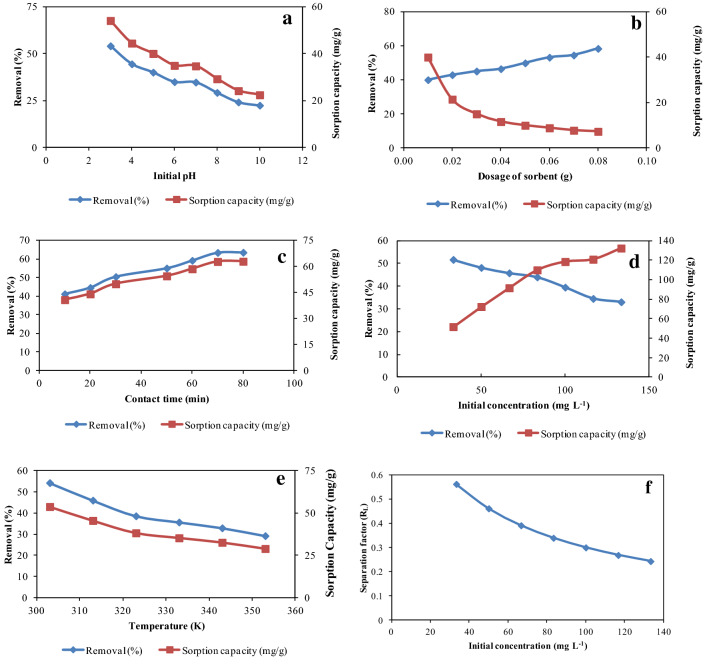
Effect of pH on removal of halosulfuron methyl employing NSP (**a**), Effect of sorbent dose on the removal of halosulfuron methyl onto NSP (**b**), Effect of contact time on the removal of halosulfuron methyl onto NSP (**c**), Effect of initial concentration of halosulfuron methyl on the removal of halosulfuron methyl onto NSP (**d**), Effect of temperature on the removal of halosulfuron methyl using NSP (**e**) Effect of initial concentration of halosulfuron methyl on dimensionless equilibrium parameter (**f**).

**Figure 6:**
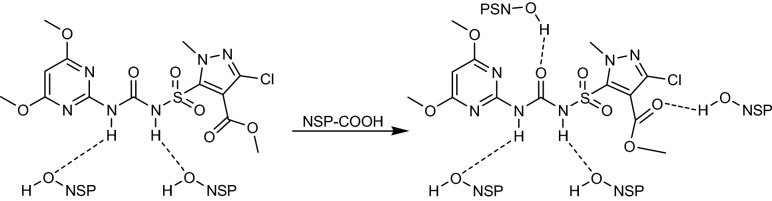
Possible interaction of halosulfuron methyl molecule with NSP.

Solution pH serves a vital function in removal of pollutants because it changes the chemistry of sorbent surface and the interaction of sorbent and sorbate molecule^45^. Consequently, the reliance of removal of halosulfuron methyl on initial solution pH was ascertained by changing pH in ranging of 3–10 with a particular initial concentration (33.33 mg L^−1^) of halosulfuron methyl. The change in removal of halosulfuron methyl with respect to pH is shown in Fig. 5a which depicts that maximum removal of halosulfuron methyl was noted at low pH but a continuous decrease in the removal was observed after increasing pH of solution. It may be inferred from this result that favorable pH to remove halosulfuron methyl is low. The possible interaction between halosulfuron methyl and NSP is given in the equation shown in Fig. 6. At low pH, protonation of the NSP surface occurs which then further interact with halosulfuron methyl molecule through van der Waals forces^46^. As a result, maximum removal of halosulfuron methyl was obtained at low pH but at high pH the interaction between halosulfuron methyl and NSP become weak due to which a continuous decrease in removal process was observed.

### Effect of sorbent dose

Along with other variables, sorbent dose has also been included in those factors which can influence the removal of pollutants^40^. To check the variation in removal of halosulfuron methyl with respect to dose of NSP, dose of NSP was changed in the range of 0.01–0.08 (gram) with a desirable concentration of halosulfuron methyl. The result is illustrated in Fig. 5b, indicating that removal percentage of halosulfuron methyl was raised with progress in sorbent dose. The surface area increases as the dose of sorbent was increased due to which more sorptive sites are accessible for interaction of sorbate molecules. Consequently, maximal removal of halosulfuron methyl was found at high dose of NSP^47^. However, sorption capacity was found to be decreased continuously with increase in dose of NSP and then the sorption capacity become constant. Such reduction in the sorption capacity with respect to dose of NSP may be ascribed to a relative amount of halosulfuron methyl in solution with regard to sorptive sites on NSP^48^.

### Effect of contact time

Time of contact also serves an essential role in removal study as it identifies time needed for maximum interaction of sorbate molecules with active sites on sorbent surface^49^. Therefore, the removal of halosulfuron methyl using NSP was studied regarding the impact of contact time by altering time in ranging of 10–80 min with particular concentration of halosulfuron methyl (33.33 mg L^−1^) and sorbent dose (0.01 g). The outcome of this factor is depicted in Fig. 5c which exhibits that percent removal of halosulfuron methyl onto NSP was raised as contact time was increased and within 70 min, equilibrium was obtained. However, no appreciable variation in the percent removal was noticed after 70 min. Such finding signifies that availability of time for the interaction of halosulfuron methyl and binding sites increases with progress in time of contact. Consequently, percent removal of halosulfuron methyl was raised with contact time but after establishment of equilibrium no prominent change in the percent removal was observed. Therefore, further removal experiments were performed at 70 min.

### Effect of initial concentration of halosulfuron methyl

Rate of sorption is strongly altered by initial concentration of sorbate^39^. Keeping the importance of the initial concentration of halosulfuron methyl in mind, concentration of halosulfuron methyl was changed in the range of 33.33–133.33 (mg L^−1^) whilst maintaining unchanged other variables. The consequence of this variable is shown in Fig. 5d which illustrates that percent removal of halosulfuron methyl onto NSP decreases constantly with raise in initial concentration of halosulfuron methyl. Literature survey demonstrates that initial concentration supplies a driving force for controlling resistance of mass transfer of sorbate molecules between aqueous phase and solid phase however such phenomenon is restricted at higher initial concentration perhaps because of occupation of accessible sorptive sites on sorbent^50^. Therefore, percent removal of halosulfuron methyl onto NSP was high at low initial concentration but was decreased continuously at higher initial concentration. Conversely, sorption capacity of NSP for halosulfuron methyl was increased with each rise in initial concentration. This performance was assigned to the fact that ratio of halosulfuron methyl molecules to binding sites of NSP was small at low initial concentration that results to small sorption capacity^51^. Nevertheless, as initial concentration was raised, this ratio was also increased and resultantly sorption capacity was found maximum at higher initial concentration.

### Effect of temperature

It has been cited in literature that sorption is initiated at interface of two phases through thermodynamics which relies on randomness of sorbate at surface of sorbent^40^. Consequently, change in temperature has significant task in designating randomness at the interface of two phases. Therefore, temperature was altered from 303 to 353 K in order to assess its impact on the removal of halosulfuron methyl onto NSP while other factors were kept unchanged. Figure 5e illustrates the variation of halosulfuron methyl removal with temperature which demonstrates that removal of halosulfuron methyl was constantly declined with rise in temperature. Such result indicates that high temperature did not favor the removal of halosulfuron methyl from aqueous media. Hence, it may be suggested that active sites on NSP involving in removal process were decreased with rise in temperature. Subsequently, removal of halosulfuron methyl was declined at high temperature. Therefore, further removal study of halosulfuron methyl was performed at room temperature.

### Kinetics study

Experimental data were examined using kinetic models to investigate sorption mechanism and rate controlling step^52^. Moreover, kinetics of the sorption study is required for the determination of sorption velocity^53^. Literature study revealed that a variety of kinetic models were tested to find out mechanism of sorption process but in current study five most commonly used models like pseudo first order, pseudo second order, Elovich, intraparticle and liquid film diffusion models were applied for prediction of kinetics of halosulfuron methyl removal from water media. Chi-square analysis (χ^2^) was used to evaluate the best fitting kinetic model by non-linear regression. The value of χ^2^ of each model was calculated and listed in Table 3. The lower value of χ^2^ suggested the best fitting of the predictive kinetic model.

#### Pseudo first order

According to pseudo first order there is a linear relationship between rates of coverage of sorptive sites to the vacant sorptive sites^54^. The nonlinear and linear form of this model is given in Table 2. The rate constant for pseudo first order kinetic, k_1_ (min^−1^) and q_e_ were computed from slope and intercept by plotting log (q_e_ − q_t_) against time (t).

**Table 2:** Sorption kinetics models and their linear and non-linear forms with corresponding plots.

Model	Non linear	Linear	Parameter	Plot
Pseudo first order	${q}_{t}={q}_{e}\left(1-{e}^{-{k}_{1}t}\right)$	$log\left[{q}_{e}- {q}_{t}\right]=log{q}_{e}- \left[\frac{{k}_{1}}{2.303}\right]t$	k_1_ (min^−1^): rate constant for pseudo first order q_e_ (mg g^−1^): sorption capacity at equilibrium q_t_ (mg g^−1^): Sorption capacity at time “t”	$log\left[{q}_{e}- {q}_{t}\right]$ vs $t$
Pseudo second order	${q}_{t}=\frac{\left({k}_{2}{q}_{e}^{2}.t\right)}{\left(1+{k}_{2}{q}_{e}^{2}.t\right)}$	$\frac{t}{{q}_{t}}= \frac{1}{{k}_{2}{\left({q}_{e}\right)}^{2}}+ \frac{1}{{q}_{e}}\left(t\right)$	k_2_ (g mg^−1^ min^−1^): rate constant for pseudo first order	$\frac{t}{{q}_{t}}$ vs $t$
Elovich	${q}_{t}=\beta ln\left(\alpha \beta t\right)$	${q}_{t}=\frac{1}{\beta }ln\left[\alpha \beta \right]+ \frac{1}{\beta }ln\left[t\right]$	α (mgg^−1^ min^−1^): rate of sorption process β (gmg^−1^): activation energy for chemical sorption process	${q}_{t}$ vs $ln\left[t\right]$
Intraparticle diffusion	${q}_{t}= {K}_{ip}{t}^{0.5}$	${q}_{t}= {K}_{ip}{t}^{0.5}+ I$	K_ip_ (mg g^−1^ min^0.5^): constant for intraparticle diffusion I (mg g^−1^): constant related with thickness of boundary layer	${q}_{t}$ vs ${t}^{0.5}$
Liquid film diffusion	–	$ln\left[1-F\right]= -{k}_{fd}\left(t\right)$	F: (q_t_/q_e_): fractional attainment of equilibrium k_fd_ (min^−1^): rate constant of liquid film diffusion	$-ln\left[ 1-F\right]$ vs $t$

#### Pseudo second order

Such model could be employed for prediction of kinetic performance of sorption occurs through chemisorption as presented by Ho and Mckay^55^. The nonlinear and linear form of this model is given in Table 2. The rate constant for pseudo second, k_2_ (g mg^−1^ min^−1^) and q_e_ were computed from slope and intercept by plotting t/q_t_ versus time.

#### Elovich model

According to this model, surfaces of sorbent are energetically heterogamous in nature and sorption occurs through chemisorption^53^. The constant parameters, α (mgg^−1^ min^−1^) and β (gmg^−1^) are the rate of sorption process and activation energy for chemical sorption process correspondingly. The magnitudes of these two parameters were computed from slope and intercept by potting q_t_ against ln(t).

#### Intraparticle diffusion model

Two scientists, namely Weber and Morris presented a model known as intraparticle diffusion which may be generally employed for the investigation of nature of rate controlling step^56^. The rate constant for intraparticle diffusion K_ip_ (mg g^−1^ min^0.5^) and I (mg g^−1^) were executed from the slope and intercept of plot q_t_ against t^0.5^. It has been given in the literature that intraparticle diffusion would be the rate determining step if plot of q_t_ against t^0.5^ is a straight line with zero intercept^57^. However, our results revealed intercept of the line is not zero which indicates that removal of halosulfuron methyl from aqueous solution did not control by intraparticle diffusion model.

#### Liquid film diffusion model

Surface diffusion plays a vital role in sorption process and to find out whether the sorption occurs through surface diffusion or not, the sorption data was fitted in this model^58^. Literature study suggest that removal of halosulfuron methyl will be determined by liquid film diffusion if graph of –ln(1-F) versus time has zero intercept^18^. However, our result indicates as shown in Table 2 that intercept of liquid film diffusion model is not zero which suggests that other kinetic models are involved in removal of halosulfuron methyl pesticide.

The rate constants of kinetic models and other kinetic constant factors were computed from their respective linear and non-linear form of kinetic models and listed in Table 3 along with correlation coefficients. An inclusive comparative study of the correlation coefficients (R^2^) of the above five kinetic models indicates that pseudo second order has highest value (0.99) which suggests that removal of halosulfuron methyl may be explained more satisfactorily by pseudo-2nd as compared other kinetic models. Moreover, the nearness of experimental (qe, exp) and calculated adsorption capacity (qe, cal) computed from pseudo second order demonstrating the fitness of model.

**Table 3:** Kinetic parameters for the removal of halosulfuron methyl using NSP.

Model	Parameter	Linear	Non linear
	q_e_, experimental (mg g^−1^)	62.88	62.88
Pseudo first order	q_e_, cal (mg g^−1^)	83.29	83.255
	K_1_ (min^−1^)	0.069	0.021
	χ^2^	–	50.205
	R^2^	0.6726	1.000
Pseudo second order	q_e_, cal (mg g^−1^)	70.422	54.859
	K_2_ (g mg^−1^ min^−1^)	0.00131	0.050
	χ^2^	–	7.694
	R^2^	0.9908	1.000
Elovich	α (mgg^−1^ min^−1^)	33.953	33.958
	β (g mg^−1^)	0.0884	5.911
	χ^2^	–	2.361
	R^2^	0.9526	1.000
Intraparticle diffusion	K_ip_	4.064	3.875
	I	27.085	27.085
	χ^2^	–	0.353
	R^2^	0.9842	1.000
Liquid film diffusion	K_fd_ (min^−1^)	0.0691	–
	Intercept	− 0.2811	–
	R^2^	0.6726	–

### Isotherm study

An isotherm is the graphical description of the relation between the quantity sorbed per unit mass of sorbent and the quantity of sorbate in solution at fixed temperature. It provides the information associated with division of sorbate between liquid and solid phases at different equilibrium concentrations^59^. Linear regression models of Freundlich, Langmuir, Temkin and D-R are used to investigate the best fitting isotherm as shown in Table 4 and isotherm parameters were calculated from the slopes and intercepts of these models as illustrated in Table 5.

**Table 4:** Sorption isotherms and their non-linear and linear forms with corresponding plots.

Isotherm	Non linear	Linear	Parameters	Plot
Freundlich	${q}_{e}={K}_{F}{C}_{e}^{1/n}$	$log\left({q}_{e}\right)=log{K}_{F}+ \frac{1}{n}log\left({C}_{e}\right)$	K_F_ (mg g^−1^): sorption capacity 1/n: intensity of sorption C_e_ (mg L^−1^): equilibrium concentration q_e_ (mgg^−1^): sorption capacity	$\mathit{log}\left({q}_{e}\right)$ vs $log\left({C}_{e}\right)$
Langmuir	${q}_{e}=\frac{{Q}_{max}{K}_{L}{C}_{e}}{1+{K}_{L}{C}_{e}}$	$\frac{{C}_{e}}{{q}_{e}}= \frac{1}{{Q}_{max}{K}_{L}}+ \frac{{C}_{e}}{{Q}_{max}}$ ${R}_{L}= \frac{1}{1+{K}_{L}{C}_{o}}$	Q_max_ (mg g^−1^): maximum monolayer sorption capacity K_L_ (L mg^−1^): constant related with sorption energy R_L_: separation factor C_o_ (mg L^−1^): Initial concentration	$\frac{{C}_{e}}{{q}_{e}}$ vs ${C}_{e}$
Temkin	${q}_{e}=\left(\frac{RT}{{b}_{T}}\right)ln{K}_{T}{C}_{e}$	${q}_{e}=RTln{K}_{T}+ \frac{RT}{{b}_{T}}ln{C}_{e}$	b_T_ (J mol^−1^): constant related with heat of sorption K_T_ (L g^−1^): Temkin isotherm constant	${q}_{e}$ vs $ln{C}_{e}$
D-R	${q}_{e}={q}_{d}exp\left(-\beta {\varepsilon }^{2}\right)$	${lnq}_{e}=ln{q}_{d}-\beta {\varepsilon }^{2}$ $\varepsilon =RTln\left[1+ \frac{1}{{C}_{e}}\right]$ $E= \frac{1}{\sqrt{2\beta }}$	β (mol^2^/kJ^2^): constant related with free energy q_d_ (mg g^−1^): maximum sorption capacity ε: Polanyi potential R (Jmol^−1^ K^−1^): gas constant T (K): temperature E (kJ mol^−1^): mean sorption energy	$ln{q}_{e}$ vs ${\varepsilon }^{2}$

**Table 5:** Isotherm constants for the removal of halosulfuron methyl using NSP.

Model	Parameter	Linear	Non linear
Freundlich	K_F_ (mg g^−1^)	12.416	12.416
	1/n	0.5406	0.550
	R^2^	0.9564	1.000
Langmuir	K_L_	0.0233	0.024
	Q_max_ (mg g^−1^)	200	200
	R^2^	0.9819	1.000
Temkin	K_T_	0.9688	− 0.631
	b_T_	52.446	52.566
	R^2^	0.9804	1.000
D-R	q_d_ (mg g^−1^)	122.547	122.547
	Β	4 × 10^–5^	2.01 × 10^–5^
	E (kJ/mol)	0.111	–
	R^2^	0.9206	1.00

#### Freundlich isotherm

This isotherm is an experimental equation and commonly manipulates to characterize multi-layer sorption on heterogeneous surface^60^. The value of 1/n of the Freundlich isotherm identifies the practicability of isotherms like favorable, unfavorable or irreversible if its value is 0 < 1/n < 1, 1/n > 1 or 1/n = 0 respectively^52^. In the current study, value of 1/n was found less than one which implies the favorable nature of the removal of halosulfuron methyl.

#### Langmuir isotherm

This isotherm signifies that process of sorption occurs in such a way that monolayer formed on a surface carrying constant number of vacant sites energetically identical to each other. The sorption energy is fixed and independent of the degree of saturation of the active site on sorbent^61^. The K_L_ is one of significant Langmuir constant which may be utilized for the evaluation of separation factor (R_L_) which demonstrates favorability of sorption process. This parameter signifies mechanism of sorption process whether it should be favorable (0 < R_L_ < 1), unfavorable (R_L_ > 1), linear (R_L_ = 1) or irreversible (R_L_ = 0). However, the current investigation indicates that magnitudes of R_L_ were found to be in the range of 0.56 to 0.24 as illustrated from the Fig. 4f which designates that removal of halosulfuron methyl is a favorable process.

#### Temkin isotherm

This isotherm explains that decreasing of heat of adsorption with respect to temperature is linear instead of logarithmical relationship. Furthermore, there is direct relationship between free energy and surface coverage^61^. The Temkin isotherm explains that decreasing of heat of adsorption with respect to temperature is linear instead of logarithmical relationship. Furthermore, there is direct relationship between free energy and surface coverage^61^. The constant parameters of Temkin isotherm are b_T_ (J mol^−1^) and K_T_ (L g^−1^) which are associated with heat of adsorption and Temkin isotherm constant respectively.

#### Dubinin–Radushkevich (D-R) isotherm

The Dubinin–Radushkevich (D-R) isotherm is considered as semiempirical equation which demonstrates that sorption occurs through pore filling mechanism. According to this isotherm, sorption process occurs in the form of multilayer by means of van der Waals which may be applied for sorption taking place through physically^62^. The constant parameters of this isotherm are β (mol^2^/kJ^2^) and q_d_ (mg g^−1^) which are associated with free energy and maximum sorption capacity respectively. The β is also related with mean sorption energy (E) (kJ mol^−1^).

The characteristics of sorption may be demonstrated by the values of mean sorption energy. It is recognized that magnitude of E for physical sorption is E < 8 (kJ mol^−1^) while for a chemical sorption its magnitude is E > 16 (kJ mol^−1^). In the current research work magnitude of mean free energy was found to be 0.111 kJ mol^−1^, which exhibits that removal of halosulfuron methyl from aqueous medium is primarily physical sorption.

The non-linear isotherm parameters and correlation coefficient were calculated using Solver add-in with Microsoft Excel program. The isotherm parameters obtained from these nonlinear equation are given in Table 5 which shown no prominent changes in the conversion of non-linear forms to their linear ones.

A thorough assessment of the correlation coefficients suggest that removal data of halosulfuron methyl was well fitted with Langmuir isotherm due to highest R^2^ value as compared to other isotherms. This result implied that a monolayer of halosulfuron methyl molecules is formed on the surface of NSP during sorption process. Maximum sorption capacity was computed from Langmuir isotherm and was found to be 200 mg g^−1^. This comparatively high sorption capacity reveals a strong interaction between halosulfuron methyl molecules and NSP.

### Thermodynamic study

Thermodynamic study was accomplished to probe the feasibility, spontaneity and mechanism of the removal of halosulfuron methyl onto NSP from aqueous media by using thermodynamic parameters like enthalpy (ΔH°), entropy (ΔS°) and free energy (ΔG°)^63^. These were determined using the following formulae:

3 $${K}_{D}=\frac{{q}_{e}}{{C}_{e}}$$

4 $$\Delta G^\circ = -RTln{K}_{D}$$

5 $$ln{K}_{D}= \frac{\Delta S^\circ }{R}- \frac{\Delta H^\circ }{RT}$$

In these equations K_D_, R and T express distribution coefficient, gas constant and the temperature respectively. The magnitudes of ΔH° and ΔS° were evaluated by plotting lnK_D_ versus 1/T and listed in Table 6. The negative ΔG° reveals that removal of halosulfuron methyl from aqueous media using NSP is a spontaneous process^64^. It has also been depicted from the table that value of ΔH° is negative suggests that removal of halosulfuron methyl is exothermic in nature. Likewise, the negative ΔH° proposed that removal of halosulfuron methyl onto NSP is physical adsorption^65^. The parameter ΔS° was found negative, which suggests a decrease in randomness at solid solution interface during the occupation of halosulfuron methyl molecules on the binding sites of NSP^66^.

**Table 6:** Thermodynamic parameters for the removal of halosulfuron methyl onto NSP.

Temperature (K)	ΔG° (kJ mol^−1^)	ΔH° (kJ mol^−1^)	ΔS° (J mol^−1^ K^−1^)
303	− 3.208	− 18.083	− 0.0497
313	− 2.449		
323	− 1.710		
333	− 1.415		
343	− 1.112		
353	− 0.637		

### Regeneration study

The regeneration study indicates only 20% of the adsorbed halosulfuron methyl was recovered from the loaded-NSP as shown in Table 7. However, the recovery efficiency was increased as the concentration of NaOH was increased and maximum recovery of halosulfuron methyl from the loaded-NSP was observed with 1.0 M NaOH.

**Table 7:** Regeneration of halosulfuron methyl loaded-NSP.

Concentration of NaOH (M)	Recovery of halosulfuron methyl (%)
1.0	40.56
0.1	32.75
0.01	20.25

## Conclusion

The purpose of the current study was to evaluate the potential of neem seed powder as an efficient biosorbent for the removal of halosulfuron methyl from water solution. The EDX, SEM, surface analysis indicate the interaction of halosulfuron methyl with the surface of neem seed powder. The pH study demonstrates that removal of halosulfuron methyl onto NSP is pH dependent and maximum removal was observed in acidic medium. Point of zero charge of NSP was found to be 6.5 which further confirm the suitable pH for the removal of halosulfuron methyl. Kinetic study demonstrated that removal of halosulfuron methyl onto NSP well describe by pseudo second order model. The isotherms results imply that Langmuir is the best isotherm model to explain the removal of halosulfuron methyl and maximum monolayer sorption capacity was found be 200 mg g^−1^. Thermodynamic parameters suggested the exothermic and spontaneity nature of the removal of halosulfuron methyl from aqueous solution. Overall outcome implies that neem seed power is an effective, inexpensive and ecofriendly sorbent for the treatment of wastewater.
